# Supplementary material for: Differential Pneumococcal Growth Features in Severe Invasive Disease Manifestations
Source: Microbiol Spectr. 2022 Jun 9;10(3):e00050-22. doi: 10.1128/spectrum.00050-22 (PMC9241771; doi:10.1128/spectrum.00050-22)
Supplement: Supplemental file 1 — Fig. S1 to S8. Download spectrum.00050-22-s0001.pdf, PDF file, 1.2 MB [file spectrum.00050-22-s0001.pdf]

Supplementary material Differential growth IPD

Differential pneumococcal growth features in severe invasive disease manifestations

Daan W. Arends, Wynand Alkema, Indri Hapsari Putri, Christa van der Gaast – de Jongh, Marc Eleveld, Jeroen Langereis, Quirijn de Mast, Jacques F. Meis, Marien I. de Jonge, Amelieke J.H. Cremers

Supplementary figure 1. Measurement insecurity margin compared to variation in cohort.

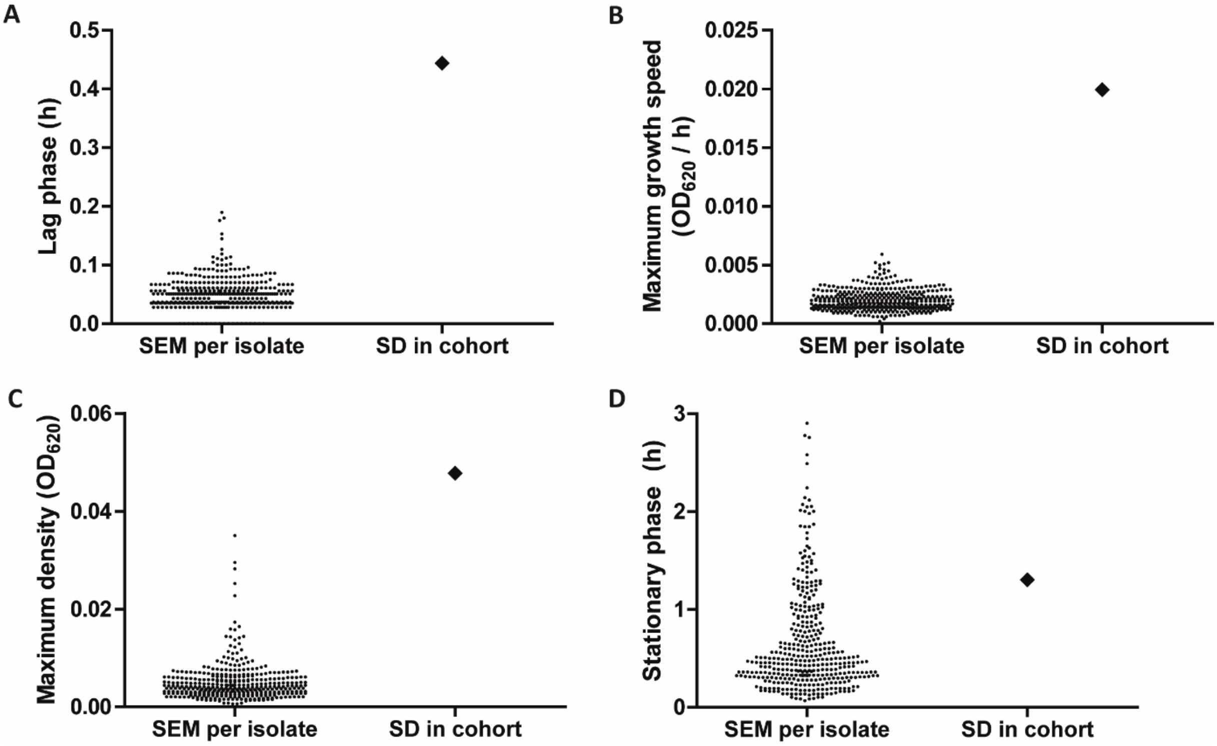

Each dot represents the standard error of the mean for 6 repeat measurements of a growth feature studied in one of the 383 clinical pneumococcal isolates. The diamond displays the standard deviation for a given growth feature across the cohort. Displayed growth features are lag phase (A), growth speed (B), maximum density (C), and stationary phase (D).
Abbreviations: SEM: standard error of the mean; SD: standard deviation; OD_{620}: optical density at 620nm wavelength.

Supplementary figure 2. Relative relatedness of growth features.

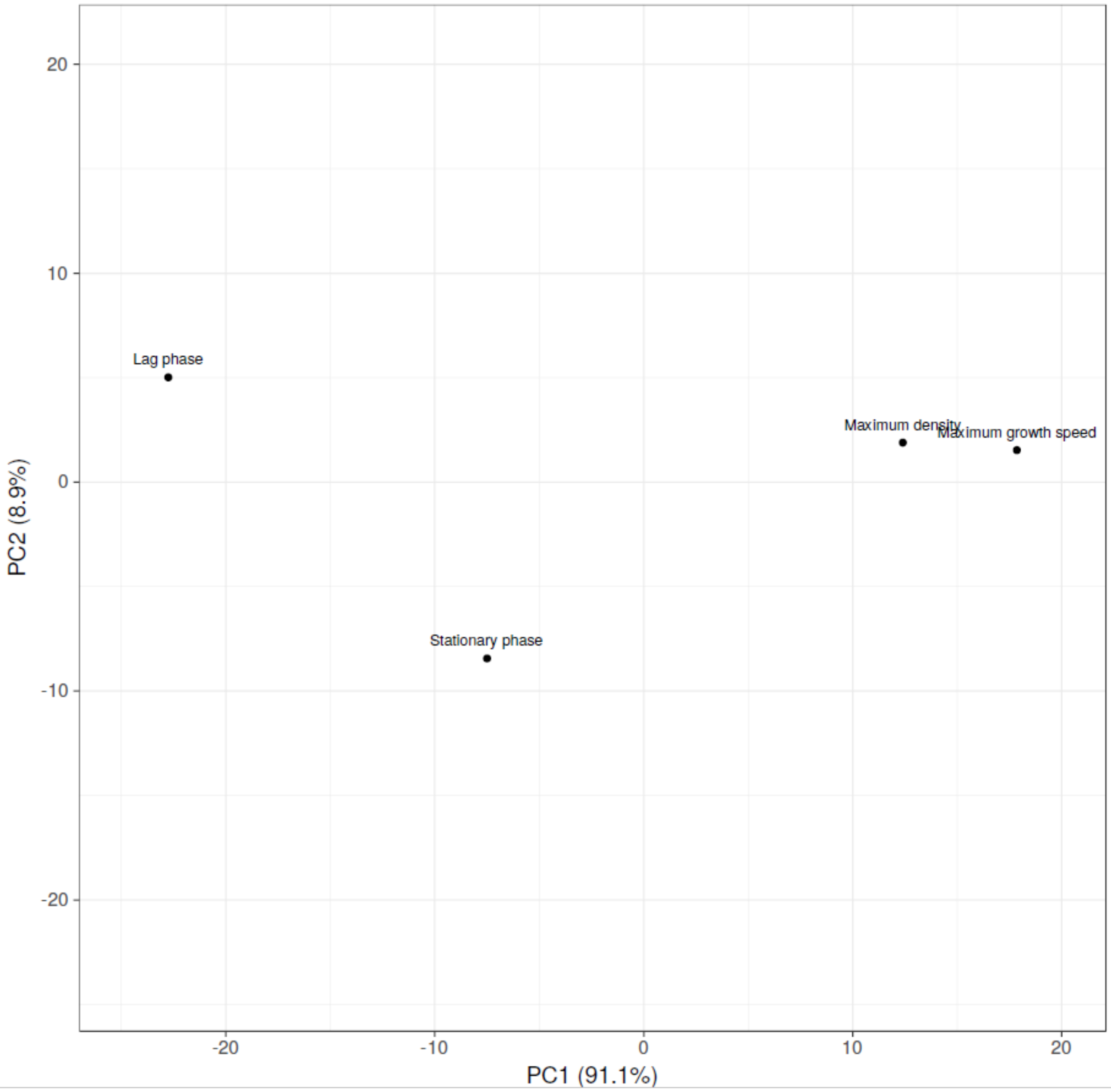

Principal Component Analysis for the 4 growth features of 383 clinical pneumococcal isolates demonstrates that the highest relative relatedness exists between maximum growth speed and maximum density. Percentages signify the proportion of variation explained by the component on that axis.

Supplementary figure 3. Correlation between growth speed and maximum density.

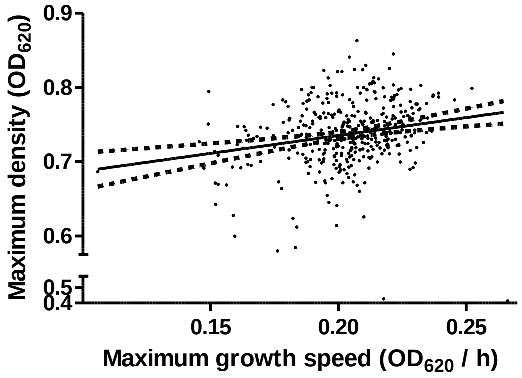

Linear regression between growth speed and maximum density for 383 clinical pneumococcal isolates demonstrates an R^2 of 0.04.

Supplementary figure 4. Growth features stratified by serotype and epidemiological relatedness to meningitis.

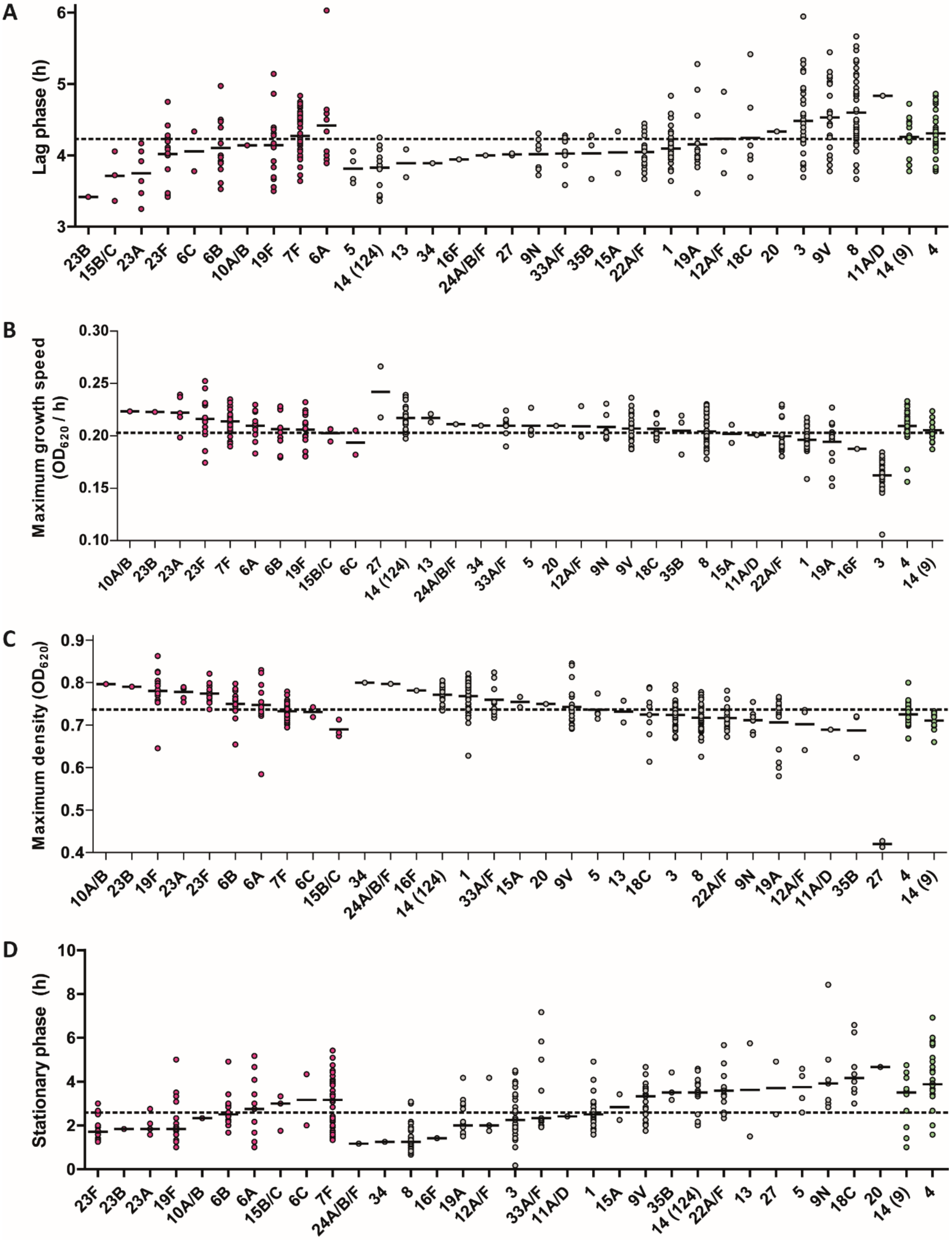

Derived growth features for 382 *S. pneumoniae* blood culture isolates sorted according to the serotypes' epidemiological propensity for causing meningitis (colors) and ranked from "pathogenic pole" onwards. Displayed growth features are lag phase (A), growth speed (B), maximum density (C), and stationary phase (D). A dashed horizontal line indicates the population mean or median respectively.

Abbreviation: OD_{620}: optical density at 620nm wavelength.

Supplementary figure 5. Effect of decapsulation on growth.

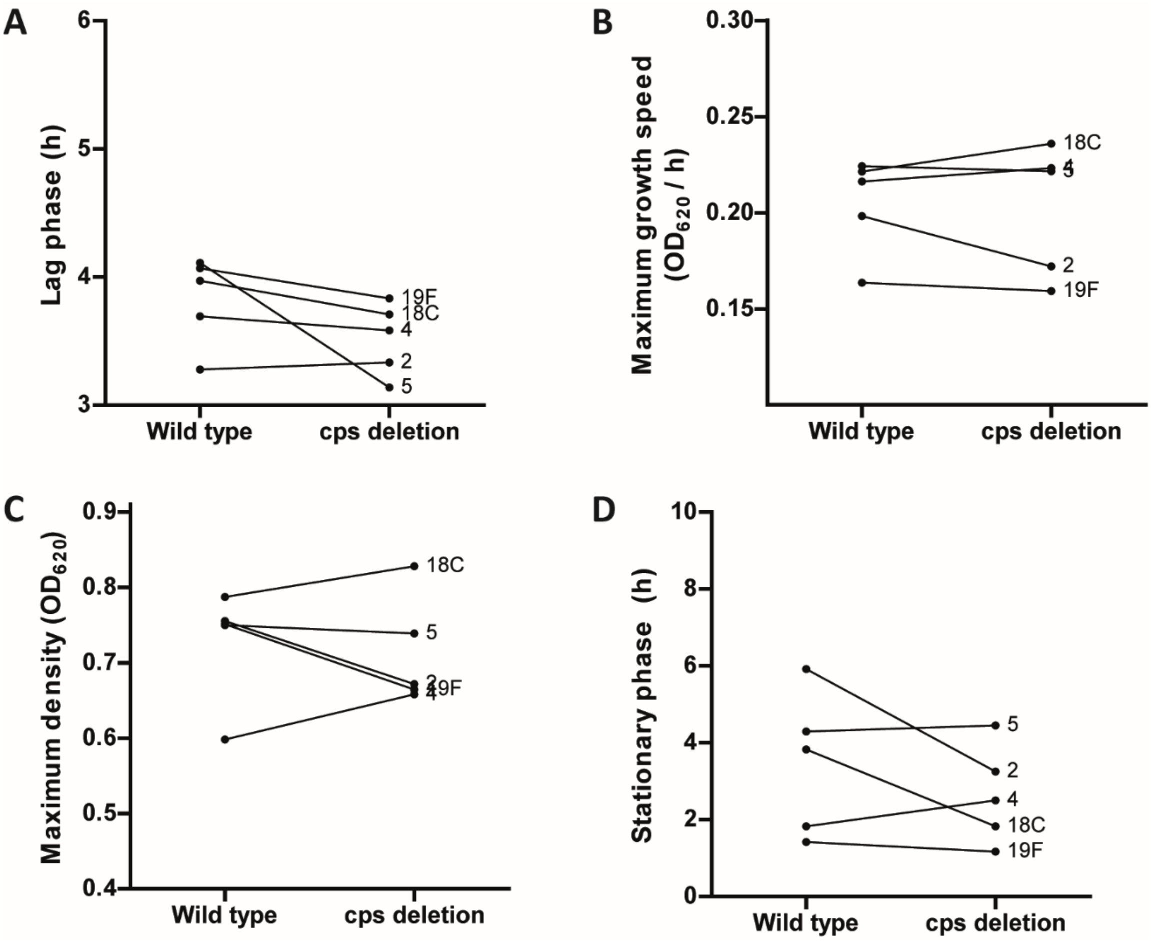

Derived growth features for 5 wild type *S. pneumoniae* strains and experimental capsular knock-out mutants. Each dot represents 6 repeat measurements for one strain. Lines in the graph connect wild type and corresponding capsular knock-out; text signifies serotypes concerned.

Abbreviations: OD_{620}: optical density at 620nm wavelength.

Supplementary figure 6. Experimental capsular switch mutants.

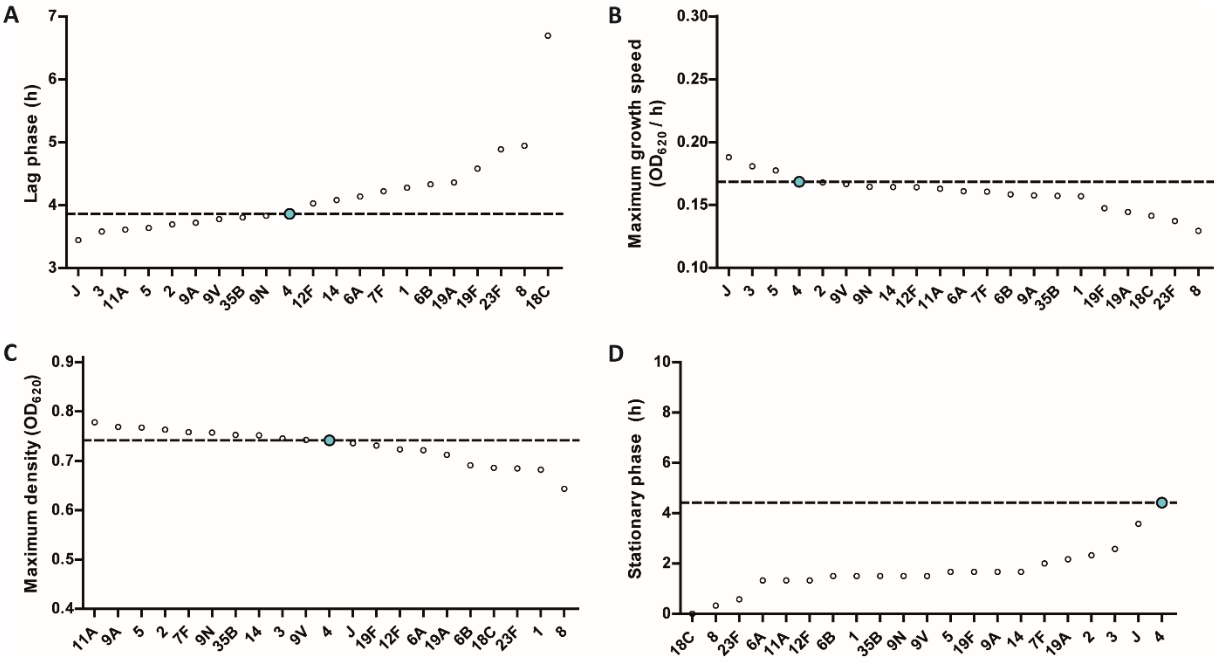

Derived growth features for experimental capsular switch mutants in a TIGR4 genetic background. Each dot represents 6 repeat measurements for one mutant. Dots are ranked from "pathogenic pole" onwards. The blue dot represents the TIGR4 wildtype with its original serotype 4 capsule expressed. Displayed growth features are lag phase (A), growth speed (B), maximum density (C), and stationary phase (D).

Abbreviations: OD_{620}: optical density at 620nm wavelength; *J*: Janus cassette, isolate without capsule locus.

Supplementary figure 7. Within-serotype differentials of empyema cases.

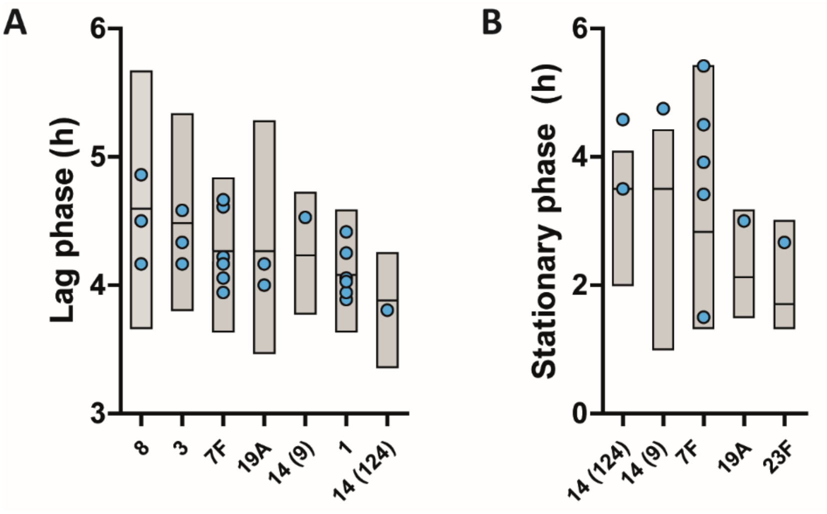

Derived growth features for *S. pneumoniae* blood culture isolates. Data are displayed for empyema cases that show marked polarity for a growth feature within their serotype. Gray boxes represent isolates from patients with solitary pneumonia caused by that serotype (from minimum to maximum, with horizontal bar at mean resp. median value), while blue dots represent isolates from patients with confirmed empyema. Displayed growth features are lag phase (A) and stationary phase (B).

Supplementary figure 8. Growth features stratified by PCV-13 serotype.

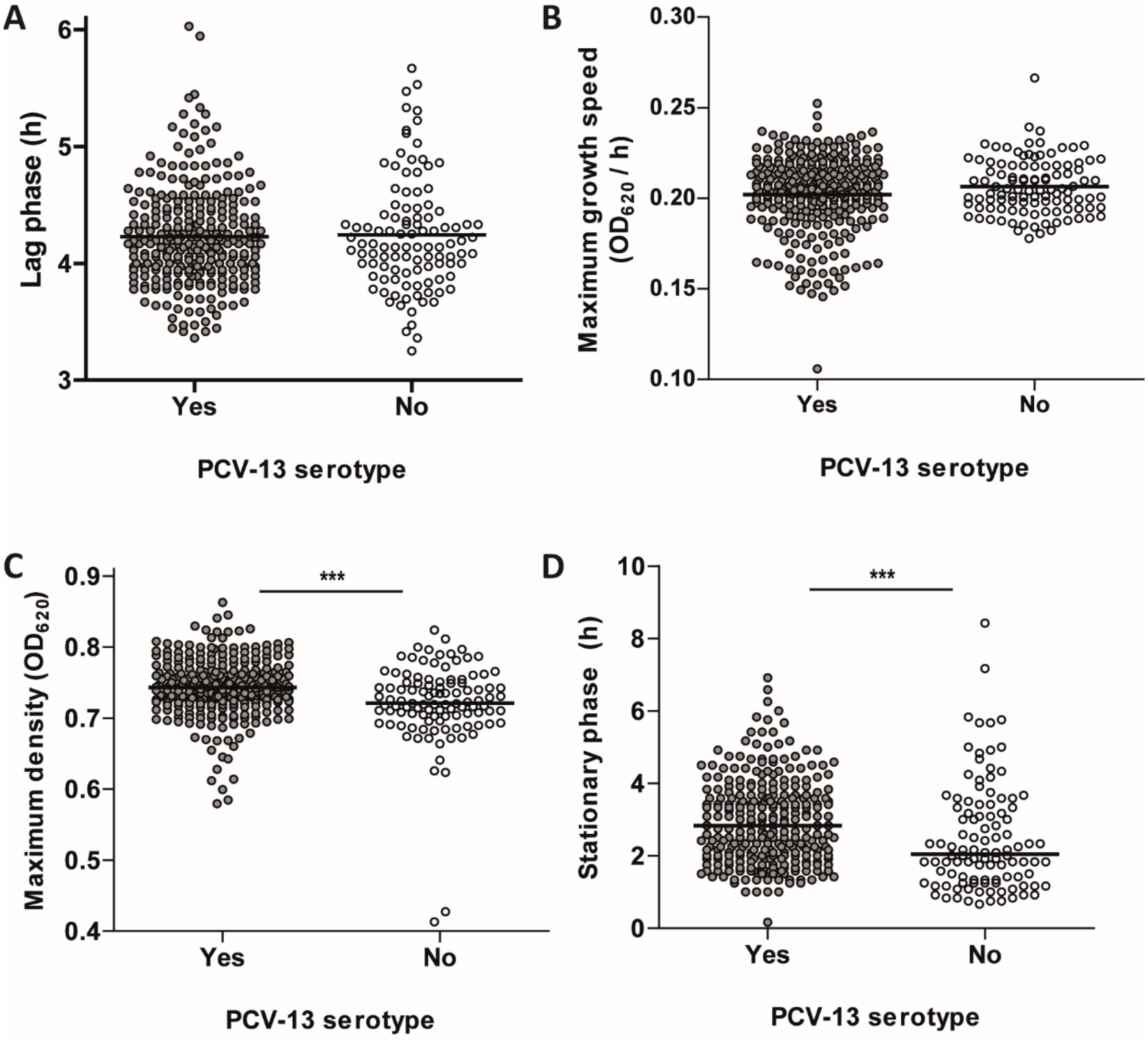

Derived growth features for 382 *S. pneumoniae* blood culture isolates sorted according to whether the serotype is targeted by the 13-valent pneumococcal conjugate vaccine. Displayed growth features are lag phase (A), growth speed (B), maximum density (C), and stationary phase (D). A horizontal line indicates the population mean or median respectively.

Abbreviations: PCV-13 serotype: serotype targeted by 13-valent pneumococcal conjugate vaccine; OD_{620}: optical density at 620nm wavelength; ***: $p < 0.0005$.
